# Supplementary material for: Optimization of spatial distribution of sports parks based on accessibility analysis
Source: PLoS One. 2023 Sep 14;18(9):e0291235. doi: 10.1371/journal.pone.0291235 (PMC10501609; doi:10.1371/journal.pone.0291235)
Supplement: S1 File — (DOCX) [file pone.0291235.s001.docx]

**Optimization of Spatial Distribution of Sports Parks Based on Accessibility Analysis**

**Questionnaire**

**Part 1 (Single-choice question)**

1. What is your gender?

(1) Male

(2) Female

2. What is your age?

(1) < 25 years old

(2) 26-35 years old

(3) 36-45 years old

(4) 46-60 years old

(5) > 60 years old

3. What is your educational background?

(1) Junior high school or below

(2) High school or technical secondary school

(3) Vocational or technical secondary school

(4) Undergraduate

(5) Graduate

4. What is your occupation?

(1) Cadres or manager

(2) Employees or workers

(3) Individual households

(4) Retirees

(5) Farmers

(6) Students

(7) Professional

(8) Others

5. What is your preferred duration of travel to the sports park for exercise?

(1) < 5 minutes

(2) < 10 minutes

(3) < 15 minutes

(4) < 20 minutes

(5) > 20 minutes

6. How do you travel to the sports park?

(1) Walking

(2) Cycling

(3) Driving

7. How often do you exercise in the sports park?

(1) Never

(2) 1-2 times per week

(3) 3-5 times or more per week

(4) More than 6 times a week

(5) not sure

8. What are your preferred time slots for exercise in the sports park?

(1) Before 8:00 a.m.

(2) 8:00 a.m. -12:00 noon

(3) 12:00 noon - 6:00 p.m.

(4) After 6:00 p.m.

9. How long do you exercise each time in the sports park?

(1) < 30 min

(2) 30 min to 60 min

(3) 1-2 hours

(4) > 2 hours

**Part 2 (Multiple-choice question)**

1. What attracts you to exercise in the sports park?

(1) Good natural environment

(2) Adequate space and facilities

(3) Close to your residence

(4) Can find peers

(5) Others

2. What are your preferred sports activities in the sports park?

(1) Basketball

(2) Badminton

(3) Table Tennis

(4) Football

(5) Tennis

(6) Golf

(7) Volleyball

(8) Walking or running

(9) Gymnastics or leisure dance

(10) Biking

(11) Traditional ethnic sports

3. What sports facilities have you used in the sports park?

(1) Basketball court

(2) Badminton court

(3) Table tennis court

(4) Football court

(5) Tennis court

(6) Golf court

(7) Volleyball court

(8) Footpath

(9) Square

(10) Biking track

(11) Fitness facilities

(12) Others

4. What problems have you encountered when doing exercises in the sports park?

(1) Insufficient quantity of space and facilities

(2) Insufficient types of space and facilities

(3) Ineffective facility distribution

(4) Delayed facility maintenance

(5) Poor environment

(6) No issues found

5. How would you like to exercise in the sports park?

(1) As a member of sports organizations

(2) With Family members

(3) With Friends

(4) Alone

(5) Others

6. What are the effects of exercise in the sports park over the past year?

(1) Development of exercise habits

(2) Mood improvement

(3) Enhancement of physical fitness

(4) Development of appropriate exercise methods

(5) Improvement of sports capability

(6) Good health

**Part 3 (Essay question)**

1. What do you think are the most severe issues to be solved related to the services provided by sports parks in the central urban area of Changsha?

2. What sports events would you like the sports park to host? Why? What are the most urgent issues to be solved?

3. What do you think the future sports park will look like?
