# Supplementary material for: Optimization of spatial distribution of sports parks based on accessibility analysis
Source: PLoS One. 2023 Sep 14;18(9):e0291235. doi: 10.1371/journal.pone.0291235 (PMC10501609; doi:10.1371/journal.pone.0291235)
Supplement: Attachment — Submitted filename: Review_PONE-D-23-12081.docx [file pone.0291235.s002.docx]

**A Study on Sports Park Layout Optimization Based on Accessibility Analysis**

Kairan Yang^1,2^, Yujun Xie^3&^, Hengtao Guo^1,2*^

Kairan Yang^1,2^，Master of sport humanisitic sociology

^1^College of Physical Education, Hunan Normal University，Hunan，410012, China

^2^Key Laboratory of Physical Fitness and Sports Rehabilitation of Hunan Province，Hunan,410012, China

E-mail address：[2496136263@qq.com](mailto:2496136263@qq.com)

Yujun Xie^3&^，Master of Urban Planning

^3^Changzhou Institute of Building Science Group Co., Ltd.

E-mail address：812392924@qq.com

Hengtao Guo^1,2*^, Professor of sports management

^1^College of Physical Education, Hunan Normal University，Hunan，410012，China

^2^Key Laboratory of Physical Fitness and Sports Rehabilitation of Hunan Province，Hunan,410012, China

E-mail address：[76926280@qq.com](mailto:76926280@qq.com)

^&^These authors contributed equally to this work and should be considered co-first authors.

Corresponding author: Hengtao Guo, E-mail address：[76926280@qq.com](mailto:76926280@qq.com)

**Abstract:** In recent years, the fairness and convenience of public sports services have been the focus of growing attention. However, there is a lack of evaluation metrics to measure the efficiency of services from a spatial perspective, such as the accessibility and convenience for residents and the layout of sports parks. This study takes a spatial perspective, with "sports park accessibility" as the theoretical entry point. The GIS network analysis method is used to analyze the related indicators of sports park accessibility (taking the sports parks in the central urban area of Changsha as an example). On this basis, spatial syntax is applied to propose sports park layout optimization strategies. The results show that there is an imbalance in population distribution, which affects the reasonable distribution of sports park. Besides, the existing sports parks in the central urban area of Changsha have a relatively low level of accessibility. Spatial syntax-based optimization strategies can improve the spatial structure of sports parks and overall enhance the accessibility.

**Keywords:** Sports Park; Accessibility; GIS Network Analysis Method; Spatial Syntax; Layout Optimization

On October 23, 2016, the Central Committee of the Communist Party of China and the State Council issued the《Healthy China 2030 Planning Outline》, which emphasized the need to coordinate the construction of public fitness facilities for all people, strengthened the construction of sports park venues and facilities, and officially launched the construction of sports parks [1]. However, due to the late start of research and the lack of theoretical support, some existing sports parks in China have shortcomings in terms of spatial layout and resource utilization, resulting in restricted service capacity[2]. On October 29, 2021, seven departments including the National Development and Reform Commission and the General Administration of Sport jointly issued the "Guiding Opinions on Promoting the Construction of Sports Parks", further emphasizing the importance of building a sound sports park system and clarifying the ideas for sports park construction[3]. Sports parks integrate sports and fitness venues with ecological gardens and environments, effectively improving the quality of life for urban residents, highlighting the city's sports genes, enhancing people's well-being, and playing a pivotal role in advancing the national strategy of Healthy China.

In recent years, more residents have been concerned about the equal and convenient access to sports parks services, and the achievement of social fairness for "eco-sports" resource sharing. The current evaluation indicators of sports parks mainly include: per capita sports park area, facility improvement, and service satisfaction[2]. However, evaluation indicators of residents' accessibility and convenience, and the spatial layout are lacking. The fair and convenient access to sports park services for urban residents is an important basis for their location and layout[4]. This study, therefore, puts forward an evaluation from the perspective of social fairness and people-oriented approach, taking accessibility as the key evaluation indicator, to explore the rationality of sports park spatial layout from a spatial perspective. It is based on the strong demand of residents for national fitness and green ecology. The study further proposes layout optimization strategies, to provide theoretical support and practical guidance for the subsequent construction of sports parks in the new era in China.

1 Sports Park Accessibility and Measurement Methods

1.1 Concept and literature review of Sports Park Accessibility

It is essential to first consider the concept of accessibility to understand the accessibility of sports parks. Accessibility is generally not only affected by spatial distance, urban layout, road network, and transportation facilities, but also by residents' attitudes and preferences[5]. In this paper, from the standpoint of spatial vision, sports park accessibility is defined as the ease or difficulty for urban residents to overcome spatial distance and travel time to participate in sports activities at nearby sports parks.

As early as the 19^th^ century, German agricultural economist Thunnen[6] discussed the classical agricultural location theory, which implicitly explored the concept of accessibility by considering transportation as the most fundamental factor. However, this content was not summarized into a concept and disseminated at the time. In 1959, American scholar Hansen [7] first proposed the definition of accessibility - the size of the interaction between nodes within the road transportation system, or the ease or difficulty of moving from one place to another. Based on Hansen's work, Dalvi [8] further refined the notion of accessibility by adding specific modes of transportation and accessible objects to consider. He defined accessibility as the ease or difficulty of getting from one location to another facility under a given mode of transportation. This has become the widely accepted concept, which is still in use today.

Domestic research on accessibility mainly focuses on theoretical aspects such as the concept, measurement methods, and influencing factors of accessibility. However, applying accessibility to the layout of sports venues, particularly sports park layouts, has been relatively underreported. Current researches on sports parks mainly focus on development paths, spatial distribution patterns, and landscape design. For example, Zhong Xiang, Jiang Chao, Li Li and Zhou Haikui [9] analyzed the shortcomings of the current sports park policies in China by leveraging Japan's successful policy models to provide multiple policy optimization pathways, including controlling construction scale, refining licensing procedures, guiding social participation, and formulating construction standards. Stanley John, Stanley Janet and Hansen Roslynne [10] took high-quality development as the starting point, exploring the potential drivers for the high-quality development of sports parks. They proposed innovative strategies such as innovative land use and management modes, coordinated internal and external development, concept dissemination, cultural diversity inclusion, and improved security service system. Sun Junbo[11] advocates an innovative city model through the integration of artificial intelligence and architecture. A range of research methods such as grid dimension analysis, spatial autocorrelation analysis, and kernel density estimation to analyze the spatial distribution characteristics of sports parks in China[12], as well as the corresponding influencing factors. Their findings revealed significant regional disparities (denser concentrations in the south and southeast, and sparser coverage in the north and northwest). These disparities were attributed to economic driving force, resource attractiveness, urban pull, transportation promotion, population support, and environmental carrying capacity [13]. The importance of sports parks as places to escape into nature and alleviate stress[14]. The designers can use garden plants to add color to sports parks, bringing a more visually pleasing environment for visitors. Furthermore, he advocated for innovative urban sports park models through the combination of nature and sports.

Currently, the selection and layout of sports parks in China often lack attention to real factors such as urban road networks and population distribution, resulting in difficulties in achieving the desired level of service[15]. By enriching the sports park evaluation system with reasonable accessibility measurement indicators and combining spatial syntax to efficiently fill service blind spots outside the reach of sports parks, it is possible to effectively meet residents' daily fitness needs and become an important means to implement the national strategy of Healthy China[16].

1.2 Sports Park Accessibility Analysis Method

The service capability of sports parks is closely related to their accessibility. The stronger accessibility allows more residents to conveniently benefit from the services provided by sports parks[17]. Ideally, ignoring various barriers to residents' travel, a shorter straight-line distance between residents and sports parks results in a shorter journey time and a higher accessibility. However, due to the complex urban road network and various road barriers, it is necessary to take into account factors such as spatial layout, urban road network, and transportation facilities apart from straight-line distances[18]. Through literature review, we identified commonly used accessibility analysis methods (Table 1). Since the road data was retrieved from the OpenStreetMap database, the network analysis method was chosen as the analysis method, considering its strong objectivity and high accuracy.

Table 1 Comparison of Accessibility Analysis Method Features

| Measurement Method | Quantitative Simplicity | Data Accessibility | Evaluation Subjectivity | Spatial Layout | Reachability Accuracy |
| --- | --- | --- | --- | --- | --- |
| BufferAnalysis | Simple | Simple | Somewhat Subjective | Lacking | Inaccurate |
| Statistical Indicators | Simple | Simple | Somewhat Subjective | Lacking | Inaccurate |
| Minimum Proximity | Moderate | Moderate | Somewhat Subjective | Lacking | Relatively Accurate |
| Cost Weighting | Moderate | Moderate | Somewhat Subjective | Present | Relatively Accurate |
| Gravity Potential | Difficult | Difficult | Subjective | Lacking | Relatively Accurate |
| Two-step Search | Difficult | Difficult | Objective | Present | Accurate |
| Network Analysis | Moderate | Difficult | Objective | Present | Accurate |

2 Regional Overview, Research Methods, and Data Sources

2.1 Study Area Overview

Changsha, the capital of Hunan Province, is in the northeastern part of the province, situated on the lower reaches of the Xiang River and the western edge of the Changliu Basin. The terrain gradually slopes downwards from the northeast and northwest towards the central part, featuring diverse landforms, well-developed surface water systems, and a typical subtropical monsoon climate[19]. The climate is gentle, with abundant precipitation, rainfall and heat occurring simultaneously, as well as distinct seasons [ ]. This study focuses on the most populous central urban areas of Changsha (Yuelu District, Yuhua District, Kaifu District, Furong District, and Tianxin District) (Figure 1).

Figure 1: Study Area

According to the 《2021 Changsha Statistical Yearbook》, the total area of the central urban area of Changsha is about 1198.2 square kilometers, with a built-up area of 357.9 square kilometers and the current sports park area of about 117.5 hectares[20]. There are a total of 13 sports parks in the central urban area of Changsha, including 4 in Kaifu District (Hongshan Sports Park, Malanshan Xiaobu Sports Park, Zifeng Sports Park, Hengdao Sports Park), 3 in Yuelu District (Yanghu Sports Park, Qingdaguge Fenglu Sports and Cultural Park, Poly·Lugu Sports Park), 3 in Yuhua District (Jinying Feixiang Sports Park, Shuangshuiwan Sports Park, Sports Culture and Leisure Park), 2 in Furong District (Dongyi Sports Park, Jiangwan Sports Park), and 1 in Tianxin District (Xiangfu Cultural and Sports Park). These sports parks are selected as the analysis objects.

2.2 Research Method

2.2.1 Network Analysis

Network analysis is a powerful tool for modelling geographic networks and infrastructure networks. Based on network topology relationships and operations research[21], it examines the spatial and attribute data of network elements and conducts multi-faceted research on network performance characteristics. It is used for finding the shortest path, optimal resource allocation, etc. This method can effectively evaluate the accessibility of sports parks, model urban road networks, and establish GIS network datasets. Under the constraints of a given transportation mode, it calculates the accessible range of sports parks within a certain resistance value.

According to field research and interviews, this research summarized the main behavioral characteristics of residents in the central urban area of Changsha when using sports parks. Most residents choose to walk or ride a bike to sports parks for physical activities if the travel time is within 0-15 minutes, and almost no one will go if it takes more than 15 minutes. Therefore, this study focuses on the accessibility of sports parks from two aspects: walking and cycling. The "Several Opinions on Accelerating the Development of Sports Industry and Promoting Sports Consumption" issued by the State Council proposed the "15-minute fitness circle". To analyze the accessibility of sports parks in the central urban area of Changsha, different time ranges of walking (4km/h) and cycling (25km/h) are divided into different accessibility levels. These results are presented in Table 2 to provide indicators for calculating the accessibility of sports parks in the mentioned area.

Table 2: Classification of Sports Park Accessibility Levels

| Travel time（min） | 0-5 | 5-10 | 10-15 |
| --- | --- | --- | --- |
| Level | one | two | three |

Four indicators are proposed to analyze the accessibility of sports parks in the central urban area of Changsha: the ratio of walkable area to the total area of sports parks, the ratio of bikeable area to the total area of sports parks, the ratio of the population served by walking to sports parks, and the ratio of the population served by biking to sports parks. By combining formulas (1) to (4), the accessibility is analyzed.

(1) Walkable Area Ratio of Sports Park = (Area within walkable range / Total research area) * 100%

(2) Bikeable Area Ratio of Sports Park = (Walkable Population Service Ratio of Sports Park / Total research area) * 100%

(3) Walkable Population Service Ratio of Sports Park = (Population within walkable range / Total research area population) * 100%

(4) Bikeable Population Service Ratio of Sports Park = (Population within bikeable range / Total research area population) * 100%

2.2.2 Space Syntax

Network analysis can objectively evaluate the overall accessibility of sports parks. It is necessary to conduct a multi-angle assessment from the aspects of both the topological structure and functional characteristics of the transportation network, in order to propose specific optimization strategies [22]. Space syntax provides multiple measurement indicators for analyzing the morphological structure of transportation networks, including connectivity, depth value, and integration. Therefore, by combining network analysis and space syntax, this study reveals the respective advantages of transportation network structure and functional characteristics, proposing an optimization strategy for the layout of sports parks.

(1) Connectivity

Connectivity represents the number of other spatial elements that intersect with the ith spatial element. The higher the connectivity, the better the spatial permeability.

(2) Mean Depth

Mean depth represents the average distance of a node to all other nodes in a network. In a road network, the mean depth can reflect the convenience of the node's location. Its calculation formula is:

 （5）

In Equation 5,represents the shortest distance between any two points in the network, and represents the total depth within the region.

(3) Integration (Integration)

Integration includes global integration and local integration. Global integration represents the topological relationship between an axis and all other axes, while local integration represents the relationship between an axis and axes within a certain range (usually three or five steps) from it. Integration can characterize the relationship between a given space and local or overall space. It is specifically defined as:

 （6）

 （7）

 （8）

 （9）

In formulas (6) to (9), $n$represents the total number of axes in the axial network;represents the integration;represents the mean depth value; Considering the number of nodes has a significant impact on the depth value,is used to normalize the integration value to eliminate its effect on integration,represents the normalized integration. Since the higher the value represents the higher accessibility, $I$, the inverse of the integration, is used as the integration indicator to evaluate accessibility. The higher the integration, the higher the accessibility.

2.3 Data Sources

2.3.1 Geographic Data

The geographic data selected in this study include road data and existing sports park data for Changsha's central urban area. Sources of road data and other linear data are mainly obtained from OpenStreetMap [23], Landsat TM remote sensing image maps, and GE satellite image maps. Subsequently, these sources were compared and interpreted along with the land-use classification maps in Changsha's general plan, and further corrected through field research. The point data of sports parks are mainly obtained through field research and actual collection in Changsha's central urban area.

2.3.2 Population Data

The population data selected in this study mainly sourced from Changsha Statistical Yearbook and Changsha City Government Portal. The statistical data from Changsha Housing and Urban-Rural Development Bureau's official website is used to extract the point information in the central urban area of Changsha[24]. After discarding incomplete data points according to the statistical yearbook, 4303 complete neighborhood point data were obtained. The population of each neighborhood point is calculated based on the detailed household information and the average population of family households in Changsha's census bulletin[25]. The spatial distribution of the population in Changsha's central urban area was obtained via simulation.

3 Analysis of Accessibility of Existing Sports Parks in the Study Area

3.1 Analysis of Accessible Area of Sports Parks

By combining the road data, neighborhood point data, population data, and other basic big data of Changsha's central urban area, the accessibility road network model is established using ArcGIS software. The network analysis method is applied to calculate the accessible area and served population of sports parks. The results are visualized in Figure 2[26].

Figure 2: Accessibility analysis of sports parks

(Left: Walking; Right: Biking)

The analysis results of the walking and cycling accessible areas of sports parks are shown in Table 3. The cumulative walking and cycling accessible areas of sports parks are 24.84 km^2^ and 223.86 km^2^, accounting for 2.08% and 18.68%, respectively. Within 15 minutes, approximately 98% of the administrative area in the central city of Changsha cannot be reached by walking to nearby sports parks, and nearly 82% of the administrative area cannot be reached by cycling. As a result, the accessible area situation of sports parks in the central city of Changsha is relatively poor.

Based on the accessibility level, the walking accessible area of Level 1 (0-5min) and the cycling accessible area of Level 1 (0-5min) are 2.59 km^2^ and 30.53 km^2^, accounting for only 0.22% and 2.55% of the study area, respectively. This indicates that the service area of sports parks in the central city of Changsha is extremely limited, providing the most convenient services to only a small portion of the population. This implies the urgent need to improve the convenience of sports park services. In addition, as calculated from Table 3, the growth value of the accessible area from Level 2 to Level 3 under cycling mode (19.31) is significantly lower than the growth value from Level 1 to Level 2 (56.48). This proves that some sports parks in the central city of Changsha are relatively close together, causing an overlap of service areas when the travel time exceeds 5 minutes. In the optimization process, further efforts should be made to reduce the travel time for residents to reach sports parks.

Table 3: Sports Park Accessibility Area Indicators

| Accessible level | Walking accessible indicators | | Cycling accessible indicators | |
| --- | --- | --- | --- | --- |
|  | Accessible area/km2 | Area ratio/% | Accessible area /km2 | Area ratio/% |
| Level 1 | 2.59 | 0.22 | 30.53 | 2.55 |
| Level 2 | 8.28 | 0.69 | 87.01 | 7.26 |
| Level 3 | 13.97 | 1.17 | 106.32 | 8.87 |
| In total | 24.84 | 2.08 | 223.86 | 18.68 |

Comparing the cycling and walking accessible areas (Table 3), the cycling accessible areas for level 1, level 2, and level 3 (30.53 km², 87.01 km², 106.32 km²) are significantly higher than those for walking (2.59 km², 8.28 km², 13.97 km²). This indicates that the road network in the central urban area of Changsha is well-developed and efficient, with cycling having an obvious speed advantage over walking. Therefore, cycling can significantly save time costs.

Comparing the coverage areas of different travel modes under the same accessibility level, the decreasing ratios of cycling to walking accessible areas for Levels 1 to 3 can be calculated as 11.79, 10.51, 7.61, respectively. This suggests that the speed advantage of cycling over walking gradually decreases with increased travel distance as road conditions become more complex. However, cycling has an overall clear advantage in short-distance travel (within 15 minutes) in the central urban area of Changsha.

3.2 Analysis of Sports Park Service Population

As can be seen in Figure 2, the population distribution in the central urban area of Changsha is uneven. Thus, relying solely on the accessible area index to judge the service level of sports parks is not appropriate. To address this issue, the author comprehensively considers the accessible area and population distribution of sports parks, and calculates the sports park service population ratio using Formula (2) and Formula (4) (Table 4) to accurately evaluate the service level of sports parks.

The statistical results presented in table 4 demonstrate the disparity of population distribution in the central urban area of Changsha. Within 15 minutes of walking, 438,900 people have access to sports parks, corresponding to 5.59% of the residential population, whereas with 15 minutes of cycling, the number increases to 4,084,500 people (58.85% of the residential population). This difference is striking compared to the findings from the analysis of accessible area ratio, which shows a 2.08% walking and an 18.68% cycling ratio.

In terms of walking, the Level 1 accessibility service population ratio for sports parks is only 0.93%, and the cumulative service population ratio is also very low where the total is less than 6%. This indicates that more than 90% of Changsha's residents cannot reach any sports park within 15 minutes, thus the walking accessibility in this study area is poor. Regarding to cycling, the cumulative service population ratio (58.85%) is acceptable, meaning that nearly 60% of residents can enjoy the services provided by sports parks within 15 minutes. The service population ratio for Level 3 cycling accessibility (28.03%) is the highest, and the ratios for other levels of accessibility are all below 25%. This indicates that a larger number of residents in the central urban area of Changsha are concentrated within the Level 3 cycling accessibility range, and the proportion of residents who can reach sports parks by cycling within 10-15 minutes is the highest.

In general, the indicators of both the sports park service population and the sports park accessible area can provide an indication of the overall accessibility and service level of sports parks in the central urban area of Changsha. However, due to the emphasis on people's well-being and the fact that sports parks serve the residents in the area, as well as the objective factors of uneven population distribution, it is more reasonable to reflect the service level using the sports park service population. Therefore, when optimizing the layout of sports parks, the focus should be on improving the sports park service population ratio. In this process, the accessible area of sports parks will also be increased to some extent.

Table 4: Sports Park Service Population Indicators

| Accessible level | Walking accessible indicators | | Cycling accessible indicators | |
| --- | --- | --- | --- | --- |
|  | Service population/ten thousand | Service population ratio/% | Service population/ten thousand | Service population ratio /% |
| Level 1 | 7.29 | 0.93 | 60.00 | 7.64 |
| Level 2 | 11.28 | 1.44 | 182.16 | 23.18 |
| Level 3 | 25.32 | 3.22 | 220.29 | 28.03 |
| In total | 43.89 | 5.59 | 408.45 | 58.85 |

4 Sports Park Layout Optimization in the Study Area

4.1 Optimization Strategies

Spatial syntax theory is an important reference for optimizing the sports park layout in this study[27]. By using depthmap to conduct an axial analysis of the spatial structure of Changsha's main urban area, the integration and connectivity of the roads are calculated and visualized via ArcGIS software (Figure 3 and Figure 4). The colors of different roads reflect the varying levels of integration (connectivity). Red roads have the best integration (connectivity), while dark blue roads have the worst one. The higher road integration indicates the stronger connectivity, which means the higher usage efficiency and the more residents passing through it. Therefore, placing sports parks around these better-integrated (connected) roads will improve their accessibility.

As one of the public facilities, sports parks should follow the principles of public facility location theory to ensure the efficient and equitable services. Due to the limited urban land, the number of sports parks should be reasonable, and their locations should be chosen in core urban areas for optimal utilization. Firstly, the core urban areas of Changsha's central city are identified based on the global road analysis (Figure 3). The global integration and global connectivity are applied to exclude poorly connected roads in the central urban area[28]. The areas with the highest density of red roads are the core urban areas (i.e., the eastern part of Yuelu District, the northern part of Tianxin District, the northern part of Yuhua District, the western part of Furong District, and the southern part of Kaifu District.

Through the optimization process, the focus should be on improving the sports park service population and considering the sports park accessibility area. By strategically placing sports parks in the core urban areas with improved road integration and connectivity, the accessibility and service level of sports parks can be significantly improved. This provides a larger number of residents in the study area with improved access to quality sports park services.

Figure 3: Global Road Analysis of Changsha's Central Urban Area

(Left image shows integration；Right image shows connectivity)

Furthermore, the optimal location for a sports park in the core urban area of Changsha need to be determined. Local integration analysis measures the integration degree of a node and its surrounding nodes within a few topological distances, reflecting the closeness between the node and its nearby nodes [ ]. By visualizing the local integration degree of Changsha's central urban area (Figure 4) and analyzing the results with three and five topological distances, the roads with the highest local integration degree in the core urban area are identified.

Additionally, the district population in Changsha's central urban area is ranked from high to low as follows: Yuelu District, Yuhua District, Tianxin District, Kaifu District, and Furong District. The spatial distribution of the population in the study area should be taken into account when planning the sports park. It is recommended that the sports park should be located in densely populated areas and near the red roads to ensure its accessibility.

Figure 4: Local Integration Degree of Changsha City Center

(Left: Three topological distances; Right: Five topological distances)

4.2 Verification of the Rationality of Layout Optimization

Based on the analysis results of the existing sports parks in Changsha city center and the spatial distribution of residents, combined with local actual planning conditions and space syntax, an optimal layout of sports parks has been proposed.

Following the optimization principle of efficiency and fairness, this layout preserves the current locations of existing sports parks while renovating or constructing four additional parks in the central part of Yuelu District, the northern part of Tianxin District, and the northern part of Yuhua District. The network analysis method is used to calculate the walkability and bikeability of sports parks in the optimized Changsha city center to verify the rationality of the layout optimization[29]. The results are visualized in Figure 5 .

Figure 5: Accessibility Analysis of Sports Parks

(Left: Walking; Right: Biking)

As shown in Figure 5, the accessible area and the served population have both increased significantly due to the increase in the number of sports parks and the optimization of their layout. The walkability and bikeability indicators of sports parks before and after optimization are calculated and compared in Table 5.

Table 5: Accessibility Indices of Sports Parks After Optimization

| Walking | Accessible level | Before optimization | | After optimization | |
| --- | --- | --- | --- | --- | --- |
|  |  | Accessible area ratio/% | Served population ratio/% | Accessible area ratio /% | Served population ratio/% |
|  | Level 1 | 0.22 | 0.93 | 0.29 | 1.42 |
|  | Level 2 | 0.69 | 1.44 | 0.96 | 2.68 |
|  | Level 3 | 1.17 | 3.22 | 1.66 | 5.76 |
|  | In total | 2.08 | 5.59 | 2.91 | 9.86 |
| Cycling | Level 1 | 2.55 | 7.64 | 3.47 | 12.27 |
|  | Level 2 | 7.26 | 23.18 | 9.68 | 33.85 |
|  | Level 3 | 8.87 | 28.03 | 7.00 | 27.70 |
|  | In total | 18.68 | 58.85 | 20.15 | 73.82 |

In terms of accessible area, the cumulative walkable area of sports parks in Changsha city center increased by 39.90% from 2.08% to 2.91%, showing a significant optimization effect on walkable area. In contrast, the cumulative bikeable area increased from 18.68% to 20.15%, with an increase of 7.87%. The optimization effect on bikeable area is relatively poor. However, according to the data after layout optimization in Table 5, the increase in bikeable area is 36.08% at level one, and 33.34% at level two. However, the bikeable area at level three was decreased by 21.08%. This is because the bikeable area of sports parks in Changsha city center had already covered the core urban areas before optimization. The sports park layout optimization strategy stipulates that new or renovated sports parks should be located in core urban areas. Although the overall increase in bikeable area after optimization is small, the level one and level two bikeable areas have significantly increased, indicating a successful optimization of the spatial structure of sports park layout in Changsha city center.

In terms of served population, the cumulative walkable service population ratio of sports parks increased from 5.59% to 9.86%, with an increase of nearly 80%. The increase in walkable service population ratio for levels one to three is 52.69%, 86.11%, and 78.89% respectively, indicating that the optimization results of sports park layout are significant under walking mode. Besides, the cumulative bikeable service population ratio reached 73.82%, and the combined bikeable service population ratio for levels one and two reached 46.12%. This means that over 70% of residents can reach a nearby sports park within 15 minutes through biking, and nearly 50% of residents can enjoy sports park services within 10 minutes. In conclusion, the optimization of sports parks under biking mode has been highly successful.

Sports parks in Changsha city center have been planned and optimized based on the local conditions and the sports needs of the residents to ensure the efficient and fair principles which are required by the location theory of public facilities[30]. Overall, after the renovation and construction of a small number of sports parks, the walking accessible area and the service population of sports parks under both walking and biking modes have been significantly improved. However, the optimization effect of bikeable area is relatively poor. This is mainly because of the high concentration of people in the central urban areas. In order to prevent resource waste, it is not feasible to blindly build sports parks outside the core areas. Considering the economic development and urban planning requirements of Changsha city center, the number of sports parks after optimization is relatively reasonable. Although the cumulative walkable service population ratio of sports parks is only 9.86%, it is already the optimal state under current conditions. Moreover, access to these sports parks is largely sufficient to meet the needs of the local residents when using bikes.

5 Conclusions and Implications

5.1 Conclusions

(1) The population distribution in the central city of Changsha is uneven, and the distribution of sports parks is unreasonable. If the population distribution were evenly distributed, the area and population accessibility indicators of the same level would be relatively similar. However, the walkable area ratio and walkable service population ratio, as well as the bikeable area ratio and bikeable service population ratio, differ significantly between districts. Besides, the population Changsha’s city center is ranked from high to low as Yuelu District, Yuhua District, Tianxin District, Kaifu District, and Furong District. The number of sports parks follows a different ranking, with Kaifu District, Yuelu District, Yuhua District, Furong District, and Tianxin District. This shows an unreasonable distribution of sports parks.

(2) The accessibility level of existing sports parks in the central city of Changsha is relatively low with only a walkable cumulative area ratio and cumulative service population ratio of 2.08% and 5.59%, respectively. This indicates that very few residents can enjoy sports park services within a 15-minute walking distance. Although the bikeable cumulative area ratio of 18.68%, and the cumulative service population ratio of 58.85% show that the number of people who can reach sports parks within 15 minutes by cycling is satisfactory, there is still possibility for improvement.

(3) Using space syntax theory, the integration and connectivity of roads in the central city of Changsha are calculated, in order to optimize the layout of sports parks. It is recommended to renovate or build four new sports parks in the central part of Yuelu District, the northern part of Tianxin District, and the northern part of Yuhua District. This will optimize the spatial structure of sports parks and improve the overall accessibility level of sports parks in the central city of Changsha.

5.2 Implications

In order to comprehensively enhance the service capacity of sports parks, expand the service scope, and increase the utilization rate of sports resources, the following suggestions are made for supporting sports venues and their service conditions:

(1) Improve transportation conditions and enhance traffic capacity. The accessibility of sports parks is closely related to road traffic capacity. Improving road traffic capacity can not only promote urban economic development, but also optimize residents' travel conditions and increase the convenience in accessing sports parks. On the one hand, the road network construction should be improved near sports parks outside the core urban areas, such as Hengdao Sports Park, to enhance the overall accessibility of sports parks in the city and expand the service range. On the other hand, for sports parks located in core urban areas with high population density, such as Qingda Valley Fenglu Sports Park, the planning of bus routes and improvement of the bus system can help alleviate residents' traffic congestion problems.
(2) Introduce professional talents and improve the service system. Sports parks are public service facilities that primarily provide high-level national fitness services, while also serving tourism, health, leisure, and having certain industrial value. Efforts should be made to continuously introduce high-level sports service, management, and operational technical personnel in Changsha, in order to build a long-term, stable, and high-quality reserve talent team for practical use in sports parks. This would allow for better services to be provided to the residents. It is also essential to persist in improving the service system, establishing a public supervision and evaluation mechanism, so that any issues encountered during sports activities can be reported and feedback on. Thereby, this enhances the scientific and democratic nature of government and relevant departments in providing services, reducing the supply imbalance of sports park resource allocation.

**Acknowledgement：**

This research was supported by the National Key R & D Program of China (Grant No. 2022YFC2010203).

**Reference:**

1.	CPC and S. Council, *Healthy China 2030 Planning Outline.* 2016.

2.	Wang, Y. and X. Li, *Research on the development of urban sports parks in China: Problems and countermeasures.* Urban Development Studies, 2018. **25(9), 120-126.**

3.	*National Development and Reform Commission (NDRC) & General Administration of Sport (GAS).* Guiding Opinions on Promoting the Construction of Sports Parks, 2021.

4.	Zhang, Z., et al., *The influence of Community Sports Parks on residents’ subjective well-being: A case study of Zhuhai City, China.* Habitat International, 2021. **117**: p. 102439.

5.	Talen, E. and L. Anselin, *Assessing spatial equity: an evaluation of measures of accessibility to public playgrounds.* Environment and planning A, 1998. **30**(4): p. 595-613.

6.	Von Thünen, J.H., *Der isolierte staat in beziehung auf landwirtschaft und nationalökonomie*. 2022: Walter de Gruyter GmbH & Co KG.

7.	Hansen, W., *How accessibility shapes land use–Journal of the American Planning Association 25-2.* DOI: <http://dx>. doi. org/10.1080/01944365908978307, 1959. **73**.

8.	Dalvi, M.Q. and K. Martin, *The measurement of accessibility: some preliminary results.* Transportation, 1976. **5**(1): p. 17-42.

9.	Zhong, X., et al., *Progress and Prospects of Research on Public Services in Sports in China: Analysis in the Context of Bibliometric Ecological Civilization and Social Networks.* Journal of Environmental and Public Health, 2022. **2022**.

10.	Stanley, J., J. Stanley, and R. Hansen, *How great cities happen: Integrating people, land use and transport*. 2023: Edward Elgar Publishing.

11.	Sun, J., et al., *Mechanical performance prediction for sustainable high-strength concrete using bio-inspired neural network.* 2022. **12**(1): p. 65.

12.	Zuo, Y., et al., *Spatial distribution pattern and influencing factors of sports tourism resources in China.* ISPRS International Journal of Geo-Information, 2021. **10**(7): p. 428.

13.	Yang, J., et al., *Delimitating urban commercial central districts by combining kernel density estimation and road intersections: a case study in nanjing city, china.* ISPRS international journal of geo-information, 2019. **8**(2): p. 93.

14.	Chiesura, A., *The role of urban parks for the sustainable city.* Landscape and urban planning, 2004. **68**(1): p. 129-138.

15.	Li, X., et al., *The six dimensions of built environment on urban vitality: Fusion evidence from multi-source data.* Cities, 2022. **121**: p. 103482.

16.	Li, B. and Y. Chen, *Tutorial for outline of the healthy China 2030 plan*. 2020, Springer.

17.	Zhang, X., H. Lu, and J.B. Holt, *Modeling spatial accessibility to parks: a national study.* International journal of health geographics, 2011. **10**(1): p. 1-14.

18.	Ma, W., et al., *Generative design in building information modelling (BIM): approaches and requirements.* 2021. **21**(16): p. 5439.

19.	Liang, J., et al., *Risk management for optimal land use planning integrating ecosystem services values: A case study in Changsha, Middle China.* Science of the Total Environment, 2017. **579**: p. 1675-1682.

20.	Bureau, C.S., *2021 Changsha Statistical Yearbook.* Changsha: Changsha Statistical Bureau, 2021.

21.	Sun, J., et al., *Machine-learning-aided prediction of flexural strength and ASR expansion for waste glass cementitious composite.* 2021. **11**(15): p. 6686.

22.	Ledewitz, S., *The Social Logic of Space*. 1991, JSTOR.

23.	Earth, G., *Google Earth satellite imagery.* 2021.

24.	Bureau, C.H.a.U.-R.D., *(n.d.).* Official Website.

25.	Government, C.C., *(n.d.).* Changsha City Government Portal.

26.	Koohsari, M.J., et al., *Public open space, physical activity, urban design and public health: Concepts, methods and research agenda.* Health & place, 2015. **33**: p. 75-82.

27.	Feng, W., et al., *Prediction of thermo-mechanical properties of rubber-modified recycled aggregate concrete.* 2022. **318**: p. 125970.

28.	Sun, Y., et al., *New insights of grouting in coal mass: from small-scale experiments to microstructures.* 2021. **13**(16): p. 9315.

29.	Sun, Y., et al., *Optimized neural network using beetle antennae search for predicting the unconfined compressive strength of jet grouting coalcretes.* 2019. **43**(4): p. 801-813.

30.	Sun, Y., et al., *Development of an ensemble intelligent model for assessing the strength of cemented paste backfill.* 2020. **2020**: p. 1-6.
