# Supplementary material for: Optimization of spatial distribution of sports parks based on accessibility analysis
Source: PLoS One. 2023 Sep 14;18(9):e0291235. doi: 10.1371/journal.pone.0291235 (PMC10501609; doi:10.1371/journal.pone.0291235)
Supplement: Attachment — Submitted filename: Response to reviewer comments 2#.docx [file pone.0291235.s004.docx]

**1 Abstract**

1.1 The term “layout of sports park” is confusing. Based on my understanding, layout of a sport park refers to the way in which different sports fields, playground, green spaces, and other sports facilities are arranged within a sport park. While in this manuscript, it mainly refers to the location / distribution of a number of parks in the city.

**Response:** Thank you for the valuable comment. The term ‘layout’ has been replaced by ‘distribution’ as suggested.

1.2 Measure efficiency of services: what is “service efficiency”? There is no definition of service efficiency and method to measure efficiency in the manuscript. I would suggest the authors to use more accurate terms (e.g. spatial accessibility) to avoid confusions.

**Response:** Thank you for the valuable comment. We agree with the reviewer that service efficiency is not a quantitative index and that’s why this study focused on accessibility and distribution of sports parks as indicators of service efficiency.

**2 Background**

2.1 Add “Background” as a section heading.

**Response:** Thank you for the valuable comment. The section heading has been added as suggested.

2.2 The author highlighted the strategic priorities of promoting the construction of sports parks at the national level. However, there is little description and definition of a sports park. For example, what is a sport park? What are the key elements of a sport park? What is the service scope? What is the purpose and benefit of constructing a sport park? Besides the policy priorities, what is the scientific importance of providing sport park in a city?

**Response:** Thank you for the valuable comment. We agree with the reviewer that more information about sports park shall be clarified. Hence, the following sentences have been added in Section 1:

- “As is known, sports can directly improve physical and mental health of human being. In 2014, “fitness for all” was officially proposed as a national strategy of China and sports and leisure activities have becoming a key part of daily life of urban citizens since then [1].”
- “A sports park refers to a specialized park with complete sports and fitness facilities for competitions and training of athletes and daily sports and leisure activities of urban citizens.”
- “As an integration of sports and leisure facilities and ecological gardens, sports parks have been playing a key role in public health and the national strategy of Healthy China by providing sports avenues to urban residents, thereby enhancing their quality of life [3, 4].”

2.3 When you say “convenient access” and “fair access”, can you define it with one or two sentence? Also, how do you measure “convenient access and fair access”? Do you mean spatial accessibility? If so, use spatial accessibility consistently to avoid confusion.

**Response:** Thank you for the valuable comment. We agree with the reviewer that ‘convenient’ and ‘fair’ are subjective terms that should be avoided in academic writing. Hence, these terms have been either removed or substituted by ‘spatial accessibility’, as suggested.

2.4 The authors take a whole paragraph to introduce current research on access to parks. However, most of the cited researches do not have a focus on spatial distribution of access to parks. I would suggest the authors use one to two sentences, instead of a whole paragraph, to summarize the previous findings on sport park policies, driving factors for the high-quality sports parks, city model, and combination of nature and sports. Please focus on literatures of “spatial distribution patterns”.

**Response:** Thank you for the valuable comment. It is true that we have not found sufficient studies discussing spatial distribution of sports parks. Hence, this part (Paragraph 2 of ‘Introduction’ in the revised manuscript) has been revised as suggested.

2.5 Please introduce “spatial syntax” briefly using one sentence in case readers may be not familiar with the term.

**Response:** Thank you for the valuable comment. The following sentence has been added into the manuscript:

“Spatial syntax refers to an analysis tool for spatial configurations and human activity patterns in buildings and urban areas. It addresses where people are, how they move, how they adapt, how they develop and how they talk about it [16].”

2.6 Table 1. First column “measurement method”. Please use lines or spaces to separate those methods from each other.

**Response:** Thank you for the valuable comment. Table 1 has been re-formatted to make it more reader-friendly.

2.7 It would be helpful to have a short paragraph at the end of background to highlight/summarize the objective and the importance of this research.

**Response:** Thank you for the valuable comment. The following sentence has been added at the end of Section 1 as suggested:

“In this study, a spatial accessibility-based method is proposed for evaluation of the distributions of sports parks. As a demonstration, the distribution of sports parks in the central urban area of Changsha, China was investigated using the proposed method by the GIS network analysis. Additionally, optimization strategies for sports park distribution (in terms of spatial distribution and overall accessibility) were developed by using spatial syntax. This study provides references and guidance to the design of sports park distribution.”

2.8 “1.1 It is essential to first consider the concept of accessibility to understand the accessibility of sports parks”. Please revise to be more succinct.

**Response:** Thank you for the valuable comment. This sentence has been removed as it is not an essential part of this manuscript. We apologize for the misunderstanding caused.

2.9 “The importance of sports parks as places to escape into nature and alleviate stress [14].” In complete sentence. Please revise.

**Response:** Thank you for the valuable comment. This sentence has been removed as it is not an essential part of this manuscript. We apologize for the misunderstanding caused.

2.10 Please check the sentence “Strengthened the construction of sports park venues and facilities, and officially launched the construction of sports parks [1]”. It is not easy to follow.

**Response:** Thank you for the valuable comment. We agree with the reviewer that this sentence is ambiguous. It has been revised as follows:

“The *Healthy China 2030 Planning Outline* issued in 2016 emphasized the significance of public fitness facilities and sports parks, and officially launched a plan for the construction of new sports parks all over China [3].”

**3 Methods**

3.1 “Regional overview” is confusing. The authors may use “Methods” as a section heading.

**Response:** Thank you for the valuable comment. The section headings have been revised accordingly, as indicated in the revised manuscript.

3.2 For the field research and interviews, how many residents were interviewed? Do you have a supplement file to report the interview results? Any literatures to support these findings?

**Response:** Thank you for the valuable comment. The results of field research and interviews have been summarized in Table 2 (shown below) in the revised manuscript. The questionnaire has been included in the Supporting Information.

**Table 2.** Preferred travel time and means of transportation to sports parks

| **Preferred travel time (min)** | **Number of interviewees** | **Preferred means of transportation** | **Number of interviewees** |
| --- | --- | --- | --- |
| 0-5 | 156 (31.2%) | Walking | 267 (53.4%) |
| 5-10 | 125 (25.0%) | Biking | 215 (43.0%) |
| 10-15 | 198 (39.6%) | Driving | 18 (3.6%) |
| 15-20 | 14 (2.8%) |  |  |
| > 20 | 7 (1.4%) |  |  |

3.3 Points data is used to represent parks. It will be helpful to provide summary information on the sizes of these parks. What is the impact of park sizes on accessibility? The use of points, instead of polygons, may be mentioned as a limitation in the discussion section.

**Response:** Thank you for the valuable comment. We agree with the reviewer that both distribution and size are key features of sports parks. Nevertheless, this study aims to clarify the accessibility of sports parks and optimization of sports park distribution. Hence, the size of these parks, which has no significant impacts on the accessibility of sports parks, is not taken into consideration.

3.4 Bikeable area ratio of sports park. Please double check the formula and review accordingly.

**Response:** Thank you for the valuable comment. We apologize for the ambiguous description. The bikeable area refers to a specific region where residents can ride a bike to a sport park nearby. It is defined as: ratio of bikeable area = (area where residents can ride to a sport park/total area of the study area) × 100%.

3.5 Methods on spatial syntax is not described in sufficient detail.

(1) Connectivity. What is spatial element in “Connectivity”? How do you define the “*i*th” spatial element?

**Response:** Thank you for the valuable comment. Spatial elements in space syntax are represented by their geometric forms and how people experience them. They are either geometrically derived or functionally defined. In this study, sports parks in the study area serve as spatial elements. The “*i*th” spatial element refers to a specific sports park to be considered.

(2) Mean Depth: define MD. What is “node” in “Mean depth”? Did you calculate d_ij_ between any two points in the whole study area or within the 15 minutes threshold?

**Response:** Thank you for the valuable comment. MD is defined as the average distance of a node to all other nodes in a network. Herein, sports parks in the study area serve as nodes. The “any two points” refer to two random points in the study area with no threshold.

(3) Integration: What is “axis” in “Integration”? How do you define number of axes? Within the whole study area or within the 15 minutes threshold?

**Response:** Thank you for the valuable comment. Axes are essential elements in the analysis of integration and they denote different lines in the graph/map; axes do not represent any specific subjects in this case. The calculation involves the whole study area with no threshold.

3.6 Authors listed remote sensing image maps and land use maps as data sources. However, the use of these data is not clearly described. Also, for point data of sports parks, is it the centre point of a park?

**Response:** Thank you for the valuable comment. The data extracted from these sources were used in this study as needed, and it is difficult to specific the utilization of each data. Herein, ‘point data of sports parks’ is relative to ‘linear data of roads’. To avoid misunderstanding, the terms of ‘linear data’ and ‘point data’ have been removed.

3.7 Please state at the beginning of the “population data” section that the data is collected for each neighbourhood point. What are the varying sizes of neighbourhoods in the city? What is the impact of using point neighbourhood, instead of polygons, on access measures?

**Response:** Thank you for the valuable comment. Herein, ‘point data of sports parks’ is relative to ‘linear data of roads’. To avoid misunderstanding, the terms of ‘linear data’ and ‘point data’ have been removed.

3.8 It is not clear how to use the three spatial syntax measures to identify the optimal location of new parks. Please describe the methods in “Methods” section, instead of “Results” section.

**Response:** Thank you for the valuable comment. The role of the three spatial syntax indexes in optimization of sports park distribution is discussed in ‘Section 4.1 Optimization strategy’. Briefly, a graph indicating connectivity, depth and integration was developed; optimization strategy and location of new sports parks are determined based on the developed graph, the road network and the buildings. Additionally, information related to methods has been moved to Section 2, as suggested.

3.9 No references were cited to support methods. Please add references accordingly.

**Response:** Thank you for the valuable comment. The following references have been cited where needed:

- Dalvi MQ, Martin KM. The measurement of accessibility: Some preliminary results. Transportation. 1976; 5(1): 17-42. doi: 10.1007/BF00165245.
- Nicholls S. Measuring the accessibility and equity of public parks: a case study using GIS. Managing Leisure. 2001; 6(4): 201-19. doi: 10.1080/13606710110084651.
- Reyes M, Páez A, Morency C. Walking accessibility to urban parks by children: A case study of Montreal. Landscape and Urban Planning. 2014; 125: 38-47. doi: https://doi.org/10.1016/j.landurbplan.2014.02.002.

**4 Results and Discussion**

4.1 Use “Results” as section heading.

**Response:** Thank you for the valuable comment. The section heading has been changed to “Results and Discussion”, as suggested.

4.2 Please focus on the report of findings. The interpretation and explanation of results could be moved to the “Discussion” section.

**Response:** Thank you for the valuable comment. This section has been revised as ‘Results and Discussion’ and focuses on both presentation and interpretation of the results obtained.

4.3 Authors stated that “As can be seen in Figure 2, the population distribution in the central urban area of Changsha is uneven.” However, there is no population information on Figure 2. Please double check.

**Response:** Thank you for the valuable comment. In Fig 2, the distribution of residential communities (grey dots) is illustrated and denser dots denote higher population density. Fig 2 has been modified so that the community distribution is more reader-friendly.

4.4 Could you please elaborate on this “Based on the analysis results of the existing sports parks in Changsha city center and the spatial distribution of residents, combined with local actual planning conditions and space syntax, an optimal layout of sports parks has been proposed.”? Do you also use local planning conditions for the optimization strategy? What is “topological distance”? It is not clear how you end up with 4 new parks and specific location of the 4 parks. It will be much helpful if the authors could describe this with details in the Methods section.

**Response:** Thank you for the valuable comment. The sentenced you mentioned is ambiguous and has been removed.

The local planning conditions of the study area are involved in terms of road network and community distribution.

Topological distance measures the structural difference between two subjects in a metric space. It is a key parameter used in analysis of local integration.

The elaboration of the quantity of new sports parks is as follows: “According to the *Guiding Opinions on Promoting the Construction of Sports Parks* issued by National Development and Reform Commission of China, one sports park can serve up to 450,000 residents [34]. As the current population in the study area is 7.85 million, a total of 17 sports parks are needed, indicating that four new sports parks shall be constructed.”

The elaboration of the locations of new sports parks is as follows: “Then, the spatial locations of new sports parks in the study area were determined. As mentioned above, sports parks shall be arranged along the roads with high integration and connectivity to maximize their accessibility. Herein, the spatial locations of new sports parks in the study area were determined based on the road network analysis shown in Fig. 3. However, global integration and connectivity do not provide sufficient information for determination of new park locations. Hence, location integration (R3 and R5, see Fig. 4) of the road network was analyzed, wherein R3 and R5 refers to three and five topological distances, respectively [35]. Specifically, roads with maximum local integration were identified and the new sports parks are located along these roads.”

4.5 What is the impact of park capacity on the optimization strategy given that population is the factor used for optimization?

**Response:** Thank you for the valuable comment. We agree with the reviewer that the capacity of each sports park may have impacts on the optimization of sports parks. Nevertheless, this study focuses on spatial distribution of sports parks and its optimization, while the capacities of all parks are assumed to be equal. In other words, the impact of park capacity on the optimization strategy is beyond the scope of this study. Future studies may involve such impacts.

**5 Discussion and conclusions**

5.1 It seems that there is no report of results on uneven population distribution. Please provide data to support your statement.

**Response:** Thank you for the valuable comment. The uneven population distribution in the study area is a fact and it is induced by various factors, including topology, history and urban planning, suggesting that results on this topic are too complicated to be discussed. Besides, the uneven population distribution is beyond the scope of this study. However, the population distribution has been considered in the optimization process: optimization strategies are developed based on maximization of areas and population with accessibility to sports parks.

5.2 The points in “5.1 Conclusions” are repeated in the “results” section. Authors could delete discussion points in the “results” section and discuss results in the “Discussion” section.

**Response:** Thank you for the valuable comment. The first paragraph has been revised, as follows:

“The distribution of existing sports parks is not rational owing to the uneven population distribution. Specifically, the quantities of existing sports parks in different districts are not consistent with the populations of these districts. The walking-accessibility of existing sports parks in the study area is low and the biking-accessibility of existing sports parks in the study area is moderate, but can be further improved. Additionally, an optimization strategy for the spatial distribution of sports parks was proposed and the results demonstrated that construction of four new sports parks can lead to a significantly improved biking-accessibility to sports parks (78.89%).”

5.3 The section “5.2 Implications” focuses on how to enhance service capacity, expand service scope, and increase utilization, which is not supported by the findings on spatial access to sports parks. Especially the second point on “Introduce professional talents and improve the service system”, which is not relevant to “spatial access to parks”.

**Response:** Thank you for the valuable comment. We agree with the reviewer that this part is not closely related to the results of this study. Hence, it has been removed.

5.4 It is important to describe the strength and limitations of this research.

**Response:** Thank you for the valuable comment. The strength and limitations of this research have been described by the following paragraph:

“This study provides a thorough investigation of walking- and biking-accessibility of sports parks in the study area, as well as a rational and effective optimization strategy for the. Nevertheless, this study has several limitations. Firstly, the characteristics of each sports park, including size, facilities and fee (if any), have not been taken into consideration. Secondly, the availability of public bikes, which may have impacts on the biking-accessibility to sports parks, have not been taken into consideration.”
