# Supplementary material for: Optimization of spatial distribution of sports parks based on accessibility analysis
Source: PLoS One. 2023 Sep 14;18(9):e0291235. doi: 10.1371/journal.pone.0291235 (PMC10501609; doi:10.1371/journal.pone.0291235)
Supplement: Attachment — Submitted filename: Reviewer comments.pdf [file pone.0291235.s003.pdf]

The manuscript examined the optimal distribution of sports parks in the central urban area of Changsha, China, using a spatial analysis approach, e.g. network analysis. Comments on each section of this work are provided below.

1 Abstract

1.1 The term “layout of sports park” is confusing. Based on my understanding, layout of a sport park refers to the way in which different sports fields, playground, green spaces, and other sports facilities are arranged within a sport park. While in this manuscript, it mainly refers to the location / distribution of a number of parks in the city.

1.2 Measure effeciency of servcies: what is “service efficiency”? There is no definition of servcie efficiency and method to measure efficiency in the manuscript. I would suggest the authors to use more accurate terms (e.g. spatial accessibility) to avoid confusions.

2 Backgound

2.1 Add “Background” as a section heading.

2.2 The author highlighed the strategic priorities of promoting the construction of sprots parks at the national level. However there is little description and definition of a sports park. For example, what is a sport park? What are the key elements of a sport park? What is the servcie scope? What is the purpose and benefit of constructing a sport park? Besides the policy prioties, what is the scientific importance of providing sport park in a city?

2.3 When you say “convenient access” and “fair access”, can you define it with one or two sentence? Also, how do you measure “convenient access and fair access”? Do you mean spatial accessibility? If so, use spatial accessibility consistently to avoid confusion.

2.4 The authors take a whole paragrah to introduce current research on access to parks. However, most of the cited researches do not have a focus on spatial distribution of access to parks. I would suggest the authors use one to two sentences, instead of a whole paragraph, to summarize the pervious findings on sport park policies, driving factors for the high-quality sports parks, city model, and combination of nature and sports. Please focus on literatures of “spatial distribution patterns”.

2.5 Please introduce “spatial syntax” brifely using one sentence in case readers may be not familiar with the term.

2.6 Table 1. First column “measurement method”. Please use lines or spaces to sepearte those methods from each other.

2.7 It would be helpful to have a short paragraphy at the end of background to highlight/ summarize the objective and the importance of this research.

2.8 “1.1 It is essential to first consider the concept of accessibility to understand the accessibility of sports parks”. Please revise to be more succinct.

2.9 “The importance of sports parks as places to escape into nature and alleviate stress[14].” In complete sentence. Please revise.

2.10 Please check the sentence “Strengthened the construction of sports park venues and facilities, and officially launched the construction of sports parks [1]”. It is not easy to follow.

3 Methods

3.1 “Regional overview” is confusing. The authors may use “Methods” as a section heading.

3.2 For the field research and interviews, how many residents were interviewed? Do you have a supplement file to report the interview results? Any literatures to support these findings?
3.3 Points data is used to represent parks. It will be helpful to provide summary information on the sizes of these park. What is the impact of park sizes on accessiblity? The use of points, instead of polygons, may be mentioned as a limitation in the discussion section.
3.4 Bikeable area ratio of sports park. Please double check the fomula and review accordingly.
3.5 Methods on spatial syntax is not described in sufficient detail.
(1) Conncetivity. What is spatial element in “Connectivity”? How do you define the “ith” spatial element?
(2) Mean Depth: define MD. What is “node” in “Mean depth”? Did you calcuate dij between any two points in the whole study area or within the 15 minutes threshold?
(3) Integration: What is “axis” in “Integration”? How do you define number of axes? Within the whole study area or within the 15 minutes threshold?
3.6 Authors listed remote sensing image maps and land use maps as data sources. However, the use of these data is not clearly described. Also, for point data of sports parks, is it the centre point of a park?
3.7 Please state at the beginning of the “population data” section that the data is collected for each neighbourhood point. What are the varying sizes of neighbourhoods in the city? What is the impact of using point neighbourhood, instead of polygons, on access measures?
3.8 It is not clear how to use the three spatial syntax measures to identify the optimal location of new parks. Please describe the methods in “Methods” section, instead of “Results” section.
3.9 No references were cited to support methods. Please add references accordingly.

4 Results

4.1 Use “Results” as section heading.
4.2 Please focus on the report of findings. The intepretation and explanation of results could be moved to the “Discussion” section.
4.3 Authors stated that “As can be seen in Figure 2, the population distribution in the central urban area of Changsha is uneven.” However, there is population information on Figure 2. Please double check.
4.4 Could you please elaborate on this “Based on the analysis results of the existing sports parks in Changsha city center and the spatial distribution of residents, combined with local actual planning conditions and space syntax, an optimal layout of sports parks has been proposed.”? Do you also use local planning conditions for the optimization strategy?What is “topological distance”? It is not clear how you end up with 4 new parks and specific location of the 4 parks. It will be much helpful if the authors could describe this with details in the Methods section.
4.5 What is the impact of park capacity on the the optimization strategy given that population is the factor used for optimization?

5 Discussion and conclusions

5.1 It seems that there is no report of results on uneven population distribution. Please provide data to support your statement.
5.2 The points in “5.1 Conclusions” are repeated in the “results” section. Authors could delete discussion points in the “results” section and discuss results in the “Discussion” section.
5.3 The section “5.2 Implications” focuses on how to enhance service capacity, expand servie scope, and increase utilization, which is not supported by the findings on spatial access to sports parks. Especially the second point on “Introduce professional talents and improve the service system”, which is not relevant to “spatial access to parks”.
5.4 It is important to describe the strength and limitations of this research.
